# Supplementary material for: Exploring the Impact of Variability in Cell Segmentation and Tracking Approaches
Source: Microsc Res Tech. 2024 Nov 16;88(3):716–31. doi: 10.1002/jemt.24715 (PMC11842944; doi:10.1002/jemt.24715)
Supplement: Supplementary file 1 — Data S1: Supporting Information. [file JEMT-88-716-s001.pdf]

Supplementary Information

Table S1: Results of Tukey's multiple comparisons test for area

**Comparison**	**Summary**	p **value**
User1 vs. User2	ns	0.9762
User1 vs. User3	****	<0.0001
User1 vs. User4	****	<0.0001
User1 vs. User5	ns	>0.9999
User1 vs. Livecyte	ns	0.1258
User2 vs. User3	****	<0.0001
User2 vs. User4	****	<0.0001
User2 vs. User5	ns	0.9338
User2 vs. Livecyte	ns	0.4745
User3 vs. User4	ns	0.8724
User3 vs. User5	****	<0.0001
User3 vs. Livecyte	****	0.3630
User4 vs. User5	****	<0.0001
User4 vs. Livecyte	****	0.6090
User5 vs. Livecyte	ns	0.0785

Table S2: Results of Tukey's multiple comparisons test for circularity

**Comparison**	**Summary**	p **value**
User1 vs. User2	ns	0.0904
User1 vs. User3	****	<0.0001
User1 vs. User4	****	<0.0001
User1 vs. User5	ns	0.9991
User1 vs. Livecyte	****	<0.0001
User2 vs. User3	****	<0.0001
User2 vs. User4	****	<0.0001
User2 vs. User5	*	0.0356
User2 vs. Livecyte	****	<0.0001
User3 vs. User4	ns	0.9988
User3 vs. User5	****	<0.0001
User3 vs. Livecyte	ns	0.3630
User4 vs. User5	****	<0.0001
User4 vs. Livecyte	ns	0.6090
User5 vs. Livecyte	****	<0.0001

Table S3: Results of Tukey's multiple comparisons test for mean gray value

**Comparison**	**Summary**	***p* value**
User1 vs. User2	ns	0.1615
User1 vs. User3	****	<0.0001
User1 vs. User4	****	<0.0001
User1 vs. User5	ns	0.9975
User1 vs. Livecyte	****	<0.0001
User2 vs. User3	****	<0.0001
User2 vs. User4	****	<0.0001
User2 vs. User5	ns	0.0562
User2 vs. Livecyte	*	0.0109
User3 vs. User4	ns	>0.9999
User3 vs. User5	****	<0.0001
User3 vs. Livecyte	****	<0.0001
User4 vs. User5	****	<0.0001
User4 vs. Livecyte	****	<0.0001
User5 vs. Livecyte	****	<0.0001

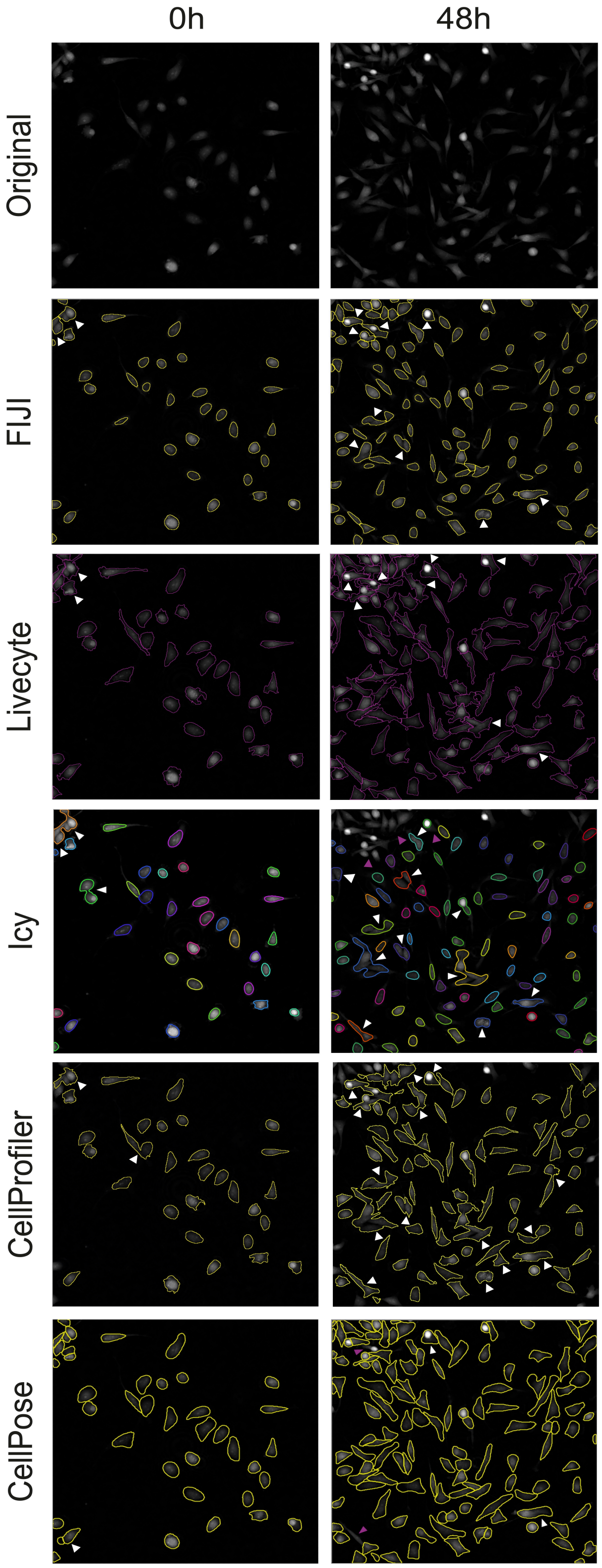

Figure S1: Segmentation results from a panel of automated segmentation software

Automated segmentation results obtained from FIJI, Livecyte, Icy, CellProfiler and CellPose on two cell images taken at 0 hours and 48 hours of a time-lapse. Instances of over- and undersegmentation are highlighted by white arrows, and cases where a cell is unidentified by automated segmentation are highlighted by pink arrows.
